# An overview of CCN4 (WISP1) role in human diseases

**DOI:** 10.1186/s12967-024-05364-8

**Published:** 2024-06-27

**Authors:** Kirti Singh, Sunday S. Oladipupo

**Affiliations:** Biotherapeutic Enabling Biology, Lilly Research Laboratories, Eli Lilly and Company, Indianapolis, IN 46225 USA

**Keywords:** CCN4, WISP1, Matricellular proteins, Extracellular matrix, Fibrosis, Cancer, Metabolism, Inflammation

## Abstract

CCN4 (cellular communication network factor 4), a highly conserved, secreted cysteine-rich matricellular protein is emerging as a key player in the development and progression of numerous disease pathologies, including cancer, fibrosis, metabolic and inflammatory disorders. Over the past two decades, extensive research on CCN4 and its family members uncovered their diverse cellular mechanisms and biological functions, including but not limited to cell proliferation, migration, invasion, angiogenesis, wound healing, repair, and apoptosis. Recent studies have demonstrated that aberrant CCN4 expression and/or associated downstream signaling is key to a vast array of pathophysiological etiology, suggesting that CCN4 could be utilized not only as a non-invasive diagnostic or prognostic marker, but also as a promising therapeutic target. The cognate receptor of CCN4 remains elusive till date, which limits understanding of the mechanistic insights on CCN4 driven disease pathologies. However, as therapeutic agents directed against CCN4 begin to make their way into the clinic, that may start to change. Also, the pathophysiological significance of CCN4 remains underexplored, hence further research is needed to shed more light on its disease and/or tissue specific functions to better understand its clinical translational benefit. This review highlights the compelling evidence of overlapping and/or diverse functional and mechanisms regulated by CCN4, in addition to addressing the challenges, study limitations and knowledge gaps on CCN4 biology and its therapeutic potential.

## Introduction

Extracellular matrix (ECM) proteins, including but not limited to collagens, fibronectins, and elastin, provide structural stability and physical framework for cellular organization across all mammalian tissues and organs. In addition to physical scaffolding, ECM proteins play a crucial role in regulating cellular processes by interacting with cell surface receptors, cytokines, growth factors and other extracellular proteins. These regulatory functions are primarily driven by a subgroup of ECM proteins known as matricellular proteins which are non-structural in nature. Matricellular proteins, such as secreted protein acidic and rich in cysteine (SPARC)-protein family, thrombospondin, periostin, osteopontin and fibulins amongst others are multidomain proteins that are secreted in the ECM and are critical for day-to-day physiological processes, including cell proliferation, migration, and adhesion [1].

In addition to other known matricellular proteins, CCN family members are highly conserved, secreted multi-modular cysteine rich proteins, composed of six homologous proteins. The first three family members namely, (i) cysteine-rich protein 61 (Cyr61), (ii) connective tissue growth factor (CTGF) and (iii) nephroblastoma overexpressed (NOV) were discovered in early 1990’s which led to the acronym CCN based on their order of discovery. CCN is also abbreviated for “Cellular Communication Network” [2]. The other three members were discovered in the late 1990’s and were associated with Wnt-1 induced signaling pathway and hence were named, (iv) Wnt-1 induced secreted protein-1 (WISP1), (v) WISP2 and (vi) WISP3. Over the past two decades, extensive research on CCN family members uncovered their diverse cellular and biological functions, designating them with alternative names listed in Table 1. The International CCN Society proposed a unifying nomenclature for the CCN family members to avoid confusion due to numerous synonymic names interchangeably used in scientific literature [3]. In 2018, the HUGO (Human Genome Organization) Gene Nomenclature Committee officially approved and adapted the new names CCN1-6 for the family members [2, 3]. For this review, these proteins are named CCN1-CCN6, in accordance with the official nomenclature recommendations.

**Table 1:** Alternative names of CCN family of proteins

*CCN1*	*CCN2*	*CCN3*	*CCN4*	*CCN5*	*CCN6*
Cyr61	CTGF	NOV	WISP1	WISP2,	WISP3
Cef10	Ecogenin	NOVH	IGFBP-rP3	CTGH-L	IGFBP-rP9
CTGF2	IGFBP8	IGFBP-rP3	Elm-1	CTGF-3	
GIG1	IGFBP-rP	IGFBP9		IGFBP-rP7	
IGFBP10	HCS24			rCOP-1	
IGFBP-rP4	HBGF-0.8			HICP	

COP-1: card-only protein; CTGF: connective tissue growth factor; Cyr61: cysteine rich 61; Elm-1: expressed in low-metastatic cells; HBGF: heparin binding growth factor; HCS: hypertrophic chondrocyte specific gene; HICP: heparin induced CCN-like protein; IGFBP: insulin-like growth factor-binding protein; NOV: nephroblastoma overexpressed; WISP: Wnt-inducible signaling pathway protein

Based on PubMed^(R)^ database, there are over 3000 publications on CCN protein family, out of which surprisingly more than 2900 studies primarily focus on CCN2 and only about 300 publications focus on CCN4, highlighting the dearth of literature and disparity towards other CCN members. Despite the overwhelming attention given to CCN protein family, relatively little is known about the role of CCN4 both in human health and diseases. Notably, the role of CCN4 has been previously studied and reviewed by researchers in a disease specific context [4, 5]. However, here, we aim to comprehensively review the growing body of literature on the diverse functions of CCN4 and its role in a vast array of pathophysiological conditions, including cancer, fibrosis, inflammatory conditions (i.e., arthritis) and metabolic disorders, including obesity and diabetes.

### CCN4 overview

**Fig. 1:**
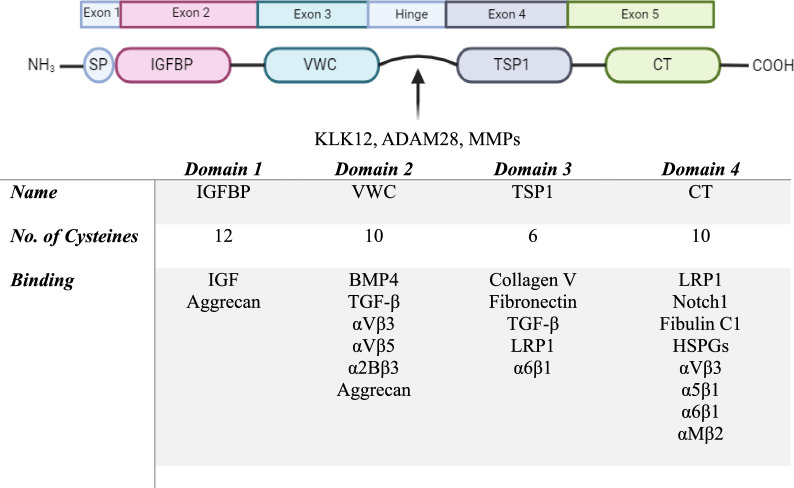
Multi-modular structure of CCN family. All the CCN matricellular proteins consist of a signal peptide (SP), insulin-like growth factor binding protein like domain (IGFBP; domain 1), von Willebrand factor C repeat (VWC; domain 2), thrombospondin-homology type 1 repeat (TSP1; domain 3) and C-terminal Cysteine knot containing domain (CT; domain 4) encoded by exon 1 to exon 5 respectively. Domain 2 and domain 4 are connected by a variable hinge region which is highly susceptible to cleavage by proteolytic enzymes such as, Kallikrein-related peptidases 12 (KLK12), a disintegrin and metalloproteinase domain-containing protein 28 (ADAM28) and matrix metalloproteases (MMPs). Domain specific total cysteine residues and binding partners are also listed in the table. The image was created with BioRender.com. IGFBP: insulin-like growth factor binding protein; VWC: von Willebrand factor C repeat; TSP1: thrombospondin-homology type 1 repeat; CT: C-terminal Cysteine knot containing domain; IGF: insulin-like growth factors; BMP4: bone morphogenic protein 4; LRP1: Low density lipoprotein receptor-related protein 1; HSPGs: heparin sulphate proteoglycans; TGF-β: transforming growth factor beta

CCN4 is the fourth member of the CCN family, commonly known as WISP1. The earliest study published on CCN4 dates back to the late 1990s where Pennica and colleagues first identified CCN4 in mouse mammary epithelial cells and its role in tumorigenesis [6]. WISP1 gene on chromosome 8q24.22 in humans encodes the 367 amino acid CCN4 protein with a predicted molecular mass of approximately 40 kDa. The murine and human CCN4 cDNA length is 1766 and 2830 bases, respectively, comprising of four introns and five exons [6, 7]. Structurally, all CCN family members are characterized by four conserved cysteine rich domains, except CCN5 which lacks domain 4. The N-terminus of CCN proteins consists of a signal peptide sequence, essential for secretory proteins, which is followed by four structural domains named based on the sequence homology to (i) insulin-like growth factor binding protein like domain (IGFBP; domain 1), (ii) the von Willebrand factor C repeat (VWC; domain 2), (iii) thrombospondin-homology type 1 repeat (TSP1; domain 3) and (iv) the C-terminal Cysteine knot containing domain (CT; domain 4) as shown in Fig. 1. The protein sequence contains 38 conserved cysteine residues, distributed across domain 1 (12 cysteine residues), domain 2 (10 cysteine residues), domain 3 (6 cysteine residues), and domain 4 (10 cysteine residues). CCN4 shares approximately 39%, 37% and 42% amino acid sequence homology to CCN2, CCN5 and CCN6, respectively [8, 9].

CCN family proteins have been shown to physically bind and interact with a plethora of multi-ligand receptors and proteins, ranging from ECM proteins (such as fibronectin [10, 11], vitronectin [12], perlecan [13], integrins [14–16], growth factors (such as fibroblast growth factors (FGFs) [17–19], vascular endothelial growth factor (VEGF) [20], bone morphogenetic protein (BMP) [21] and transforming growth factor (TGF-β) [21], proteoglycans (such as heparan sulfate proteoglycans (HSPG) [22–24], aggrecan, decorin and biglycan [25] and low-density lipoprotein receptor-related proteins (LRP) [26, 27], cation-independent mannose-6-phosphate [28], Neurogenic locus notch homolog protein (Notch) [29], and receptor activator of nuclear factor kappa B (NF-κB) (RANK) [9, 30, 31]. Despite abundance of binding partners available for CCN family of proteins, CCN4 has only been shown to interact with integrins [32, 33] and small leucine rich proteoglycans, such as decorin and biglycan [25], and domain specific binding sites that facilitate these interactions remain unknown, owing to the bias towards other CCN members, particularly CCN1 and CCN2.

Post-translational modifications (PTMs) play a crucial role in structural and functional characteristics of a protein. With respect to the CCN family, four potential N-glycosylation sites have been identified in CCN4 [6]. Similar glycosylation patterns have also been observed in CCN2, particularly at asparagine 28 and 225 leading to either 36 kDa or 38 kDa molecular weight that appears as a double band upon immunoblotting [34, 35]. Furthermore, researchers have also demonstrated that the glycosylated versus non-glycosylated ratio of CCN3 profoundly influences the cell proliferative, migrative and invasive properties of chondrosarcoma cell line Jeg3 [36]. Differences in the glycosylation patterns between normal and cancerous cells have also been reported, highlighting its functional significance in disease pathologies [37]. Besides glycosylation, O-fucosylation (O-linked fucose modification) has been recently identified in the TSP domain 3 of CCN2 protein [38]. Overall, these PTMs could be exploited to modulate CCN4 function; yet the PTM analysis in CCN family remains in its infancy, and more research is needed to shed more light on its clinical significance.

**Fig. 2:**
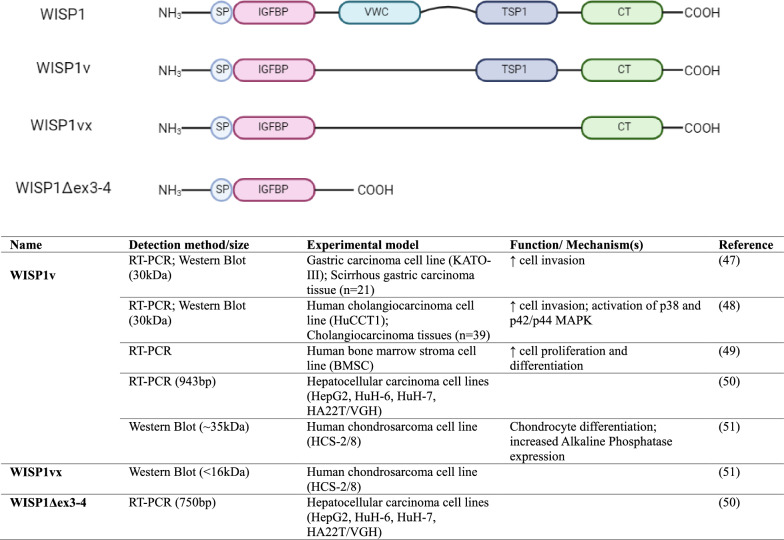
Functional effects of CCN4 Truncated variants. The image was created with BioRender.com

As previously mentioned, CCN genes contain four introns and five exons. Each of the exon codes for each domain in the structure. The N-terminus signal peptide is encoded by exon 1, whereas domain 1–4 are encoded by exon 2–5, respectively. From the evolutionary perspective, the order of CCN domains remains highly conserved and are connected through a linker. The linker connecting domain 1–2 (3aa) and domain 3–4 (9aa) in CCN4 structure are relatively short compared to domain 2–3 hinge (27aa) which makes it susceptible to proteolytic cleavage by protease enzymes, such as matrix metalloproteases (MMPs) generating truncated versions of the protein. The central variable linker has been shown to be targeted by MMP1, MMP3, MMP 7, MMP9, MMP13, MMP14 along with other peptidases, such as disintegrin and metalloproteinase domain-containing protein 28 (ADAM28), Kallikrein-related peptidases 12 (KLK12), plasmin and elastase [39–42]. Experimentally, the protease mediated degradome pattern has been extensively elucidated for CCN1, CCN2, CCN3 and CCN5 [39, 43–45]. While the cleavage sites for CCN4 remain undiscovered, researchers have identified three truncated versions in biological samples as shown in Fig. 2. In addition, the CCN4 mRNA is also subjected to alternative splicing, which can result in truncated variants of proteins, lacking one or more domains [46]. In 2001, Tanaka and colleagues reported a novel truncated CCN4 variant (Mol. Wt. 30 kDa), commonly known as WISP1v lacking domain 2 or VWR module as a product of alternative splicing in human scirrhous gastric carcinoma tissue [47]. Stable transfection of this variant increased gastric carcinoma cell migration up to five-fold as compared to full length CCN4 in a co-culture Boyden Chamber. Later in 2003, they also detected CCN4 truncated variant, WISP1v in human invasive cholangiocarcinoma tissues [48]. Further, in 2008, Inksonand colleagues at the National Institutes of Health (NIH) detected a similar CCN4 spliced variant lacking exon 3 in human bone marrow stromal cells (hBMSC) using RT-PCR, which they referred to as WISP1va [49]. In 2004, another truncated variant of CCN4, lacking domain 2, 3 and 4, also known as WISP1Δex3-4 was reported in 4 different human hepatocellular carcinoma cell lines (HuH-6, HuH-7, VGH and HepG2) in conjugation with full length and WISP1v. WISP1Δex3-4 variant only contains IGFBP/ domain 1 due to the alternative splicing of mRNA. The transcript contains exon 1, 2 and 5, however the conjugation of exon 2 and 5 causes a frame shift at residue 117, resulting in a premature stop of the reading frame and hence only translating to domain 1 [50]. Similarly, WISP1Δex3-4 truncated variant was also detected in chondrocytes derived from human chondrosarcoma cell line (HCS-2/8) and rabbit growth cartilage [51]. Although the functional significance of full length CCN4 has been extensively demonstrated, the domain specific activity remains unknown till date despite the detection of CCN4 truncated versions in human tissues.

Meanwhile, emerging evidence in the literature suggests that CCN4 can in turn modulate MMP expression. CCN4 treatment for 24 h in human chondrosarcoma cell line, JJ012 increased MMP2 expression in cell lysate and supernatant, detected at both protein and transcript level utilizing western blot and qPCR [33]. Further, CCN4 has also been shown to promote cell motility by upregulating MMP2 and MMP9 expression in human osteosarcoma cell line, U2OS as pretreatment with selective MMP2 and MMP9 inhibitors abrogated CCN4 induced wound healing and migration [32]. In addition, stimulation of murine macrophages (RAW 264.7), primary human chondrocytes and synovial cells with CCN4 (1.0 µg/ml) for 24 h upregulates MMP3 and MMP9 expression [52]. Also, CCN4 drives MMP2 and MMP9 expression in murine primary renal tubular epithelial cells as shRNA mediated CCN4 knockdown decreased MMP2 and MMP9 [53]. Finally, CCN4 has also been shown to upregulate the expression of MMP1, MMP2, MMP3, MMP9 and MMP14 in vein smooth muscle cells via β-catenin mediated pathways. CCN4 mediated MMP9 induction is partly due to activator protein-1 (AP-1) [54].

The control exercised by CCN4 on the MMPs can drive cell motility via two distinct, though parallel mechanisms. The observations derived from literature unveil that CCNs can indirectly influence cell motility by upregulating MMPs, which drives ECM degradation, facilitating cell migration and invasion [55]. Another direct mechanism by which CCN4 can influence cellular processes could be through MMP-dependent self-regulation, where CCN4-induced MMP can in turn act on CCN4 itself to generate different truncated versions of the protein. Depending on the tissue expression profile of MMP subtypes and their corresponding CCN4 cleavage sites, these truncated variants can possess similar and/or unique functional signature as compared to the full length CCN4 protein. Further, alterations in CCN4 expression profile have been observed in a plethora of diseases, ranging from cancer [56], liver fibrosis [57], idiopathic pulmonary fibrosis (IPF) [58–60], obesity and type 2 diabetes mellitus [61, 62], amongst others. The CCN-MMP interplay can continue to regulate one another, leading to a vicious positive-feedback cycle which can potentially aggravate the underlying condition by generating truncated CCN4 which could be responsible for diverse cellular functions. Taken together, CCN4 mediated regulation of MMPs is a highly complex, expression dependent, cell type and tissue-specific mechanism which yet remains underexamined and warrants further investigation.

In humans, CCN4 expression has been confirmed in various organs, such as lung, heart, kidney, pancreas, placenta, brain, small intestine, ovaries, and skeletal muscle, amongst others [6]. Within the organs, the expression is cell type specific. For example, CCN4 is mainly expressed in fibroblasts (lung [63], liver [57], heart [64]), epithelial cells (lung) [60], cardiomyocytes (heart) [65], neurons [66], microglia (brains) [67], chondrocytes [33], osteoblast (bone) [32] and many more. The diverse expression profile endows CCN4 protein with pleotropic functions in a tissue and cell specific manner. CCN4 plays a crucial role in cell proliferation, migration, adhesion, wound healing and repair in embryogenesis, fibrosis, tumorigenesis, osteoarthritis, etc. [68]. In addition, differential expression of CCN4 has been attributed to numerous diseases, which can be utilized as a prognostic biomarker.

### CCN4 in cancer

Cancer represents a global health issue with increasing cases every year [69]. According to the national cancer society, cancer is the second leading cause of death, with approximately 1 in 3 people suffering in the US. Recent technological advances in cellular and molecular biology have opened endless avenues for the development of targeted anti-cancer treatment, ranging from stem-cell therapy, gene therapy to targeted precision therapy [70]. Differential gene expression and novel biomarker discovery efforts help identify promising druggable targets with potential therapeutic benefits in the clinic. CCN4 is one such recently identified protein which plays a crucial role in inflammation and tumorigenesis [71].

Over two decades, numerous studies have investigated the role of CCN4 in tumor microenvironment, however the ambiguous expression profile and paradoxical functional outcomes of CCN4 in various cancers makes it a challenging yet controversial target. Generally, CCN4 dysregulation and atypical expression profile have been linked in a range of pathophysiological conditions, such as fibrosis, diabetes, obesity etc. [57, 72]. Similar aberrant expression profile has been observed in various cancer types as well. For instance, CCN4 is upregulated in a vast array of cancer tissue specimens compared to healthy controls, such as lung cancer [73], ovarian cancer [74], colon cancer [75], gastric cancer [76], breast cancer [77] and esophageal squamous cell carcinoma [78], among others. CCN4 is also characterized as an oncogene, that promotes tumor progression by positively regulating pro-oncogenic cellular functions, like cell proliferation, migration, and invasion. In addition, higher CCN4 protein levels were associated with low survival rate in cancer patients [79–81]. In contrast, others have reported tumor suppressive nature of CCN4, promoting cellular apoptosis whilst inhibiting cell growth, migration, invasion, and metastasis. Many researchers have also reported CCN4 downregulation in breast cancer [82, 83], liver cancer [84] and skin cancer [85]. The discrepancies could also be attributed to other confounding variables, such as patient family-history, age, gender, co-morbidities, treatment regimen, cancer-type, cancer-stage, tumor size etc., however in-depth analysis is required before establishing any correlation. Rather than simply distinguishing all diseased patients as either high or low expressers, some studies also segregate the diseased cohort into two groups, CCN4 low and high expressing patients based on the expression profile [79–81]. The spatial and temporal tumor heterogeneity leads to a complex dynamic cellular state which can also govern the distinct CCN4 expression patterns. A summary of the functional and mechanistic effects of CCN4 on diverse tumor types, along with the expression patterns is provided in Table 2.

**Table 2:** Expression and functional effects of CCN4 in cancers

Cancer type	CCN4 expression	Experimental model	Functional effect of CCN4	Mechanism involved	References
Breast cancer	Upregulated	Human breast cancer tissue (n = 44)			[101]
	Upregulated	Breast cancer cell lines (SK-BR-3, MCF7)	↑ cell proliferation, migration, and invasion		[102]
	Upregulated	Breast cancer cell lines (MCF-7, MDA-MB-231) Tumor xenograft model	↑ cell proliferation, migration, invasion, EMT and cancer growth	Blocked tumor suppressor gene, NDRG1, filamentous-actin remodeling and polarization; Increasing N-cadherin, SNAIL, β-catenin	[77]
	Upregulated	Human breast cancer tissue (n = 520) Human breast cancer cell lines (BT474, SKBR3, MDA-MB-231, 184A1, BT474, and SKBR3)	↓ type 1 mediated cytotoxic immunity and ↑ type 2 immunity	CCN4 suppresses type-1 cell mediated immunity by IL12	[103]
	Downregulated	Human breast cancer tissue (n = 120)			[82]
	Downregulated	Human metastatic breast cancer tissue (n = 85)			[83]
	Upregulated	Human breast cancer tissue (n = 356) Public database			[104]
	Upregulated	Melanoma cell lines (4T1, 67NR, B16F10) Murine breast cancer cell lines (MDA‐MB‐231, Ep5)	↑ cell invasion and metastasis CCN4 does not affect cell proliferation	Remodeling collagen architecture and collagen linearization	[105]
	Upregulated	Human breast cancer tissue Murine mammary tumor cell line (EO771)	↑ cell proliferation, migration, and invasion	CCN4 increased TGF-β, SNAIL, MMP9 and Runx2 expression	[106]
Chondrosarcoma	Upregulated	Human chondrosarcoma cell lines (JJ012, SW1353, HS18.90) Human chondrosarcoma tissue	↓cell migration CCN (shRNA) KD: ↓ migration	CCN4 increase MMP-2 expression via integrin α5β1/FAK/MEK/ERK/NF-κB pathway	[33]
Colon cancer	Upregulated	Human colon cancer tissues (n = 82) Colon cancer cell lines (LOVO, HCT-116, SW620, SW480, RKO) Tumor xenograft model	CCN4 (shRNA) KD: ↓ tumor growth, cell proliferation, invasion and ↑ apoptosis	CCN4 (shRNA) KD increases E-cadherin and decreases MMP2, MMP9, ICAM-1, VCAM-1, VEGF-C	[75]
	Upregulated	Human colon cancer tissue (n = 26) Human colon cancer cell lines (SW48s0, SW620, HCT-8, HCT-116) Normal colon cancer cell line (NCM460)	↑cell proliferation, migration, and invasion	RNA AFAP1-AS1 and miR-195-5p modulate CCN4 expression and function	[107]
Esophageal squamous cell carcinoma	Upregulated	Esophageal cancer tissue (n = 105) Esophageal cancer cell lines (TE1/4/6/8/9/10/11/14/15)	↑ cell proliferation		[108]
	Upregulated	Esophageal cancer tissue (n = 50) Esophageal cancer cell lines (KYSE-30. KYSE-150, KYSE-180) Tumor xenograft model	↑radio-resistance CCN4 (shRNA) KD: ↑ radiosensitivity	CCN4 activate anti-apoptotic PI3K and γ-H2AX	[78]
	Upregulated	Esophageal cancer tissue (n = 33) Esophageal cancer cell lines (KYSE-410, KYSE-150, TE10/11/13) Esophageal epithelial cell line (HET-1A)		CCAT-2 regulate CCN4 expression	[109]
Gastric cancer	Upregulated	Human gastric cancer tissue (n = 69) Human gastric cancer cell lines (MKN28, MKN45, HGC-27, SGC7901, BGC823) Human gastric mucosal epithelial cell line (GES-1) Tumor xenograft model	CCN4 (siRNA) KD: ↓ cell proliferation, colony formation and ↑ apoptosis	CCN4 (siRNA) KD decreases phosphorylation of Akt and mTOR	[76]
Glioblastoma	Upregulated	Human glioblastoma tissue (n = 40) Human astrocyte cell line Human glioma cell lines (SHG-44, U-251, U-373MG)	CCN4 (shRNA) KD: ↓ cell proliferation, migration, invasion, tumor growth and ↑ apoptosis	CCN4 (shRNA) KD increases p53, E-cadherin and decreases ERK, c-myc, cyclin D1, vimentin, and MMP9 expression	[110]
	Upregulated	Human glioblastoma tissue Patient derived primary human glioblastoma cell lines Tumor NSG-xenograft model Public database	CCN4 (shRNA) KD: ↓ cell proliferation, tumor growth and tumor associated macrophage	CCN4 activates Akt phosphorylation via integrin α6β1	[87]
Laryngeal cancer	–	Laryngeal squamous cancer tissue (n = 74) Laryngeal squamous cancer cell line (Hep-2)	↑ glucose uptake, cell growth, survival and ↓ cell death CCN4 (siRNA) KD: ↓ lactate production, cell survival and ↑ chemo-sensitivity	CCN4 increase GLUT1 expression via YAP1/TEAD1 signaling pathway	[111]
	Upregulated	Laryngeal cancer cell lines (TU212, TU686) Bronchial epithelial cell line (16HBE)	CCN4 (siRNA) KD: ↓ cell proliferation and ↑ apoptosis	miR-384 expression is significantly downregulated. miR-384 directly inhibit CCN4	[112]
Liver cancer	–	Human hepatocellular carcinoma cell lines (HepG2, HuH-6, HuH-7, HA22T/VGH)			[50]
	Downregulated	Human hepatocellular carcinoma tissue (n = 132) Human hepatocellular carcinoma cell lines (HuH-7, HCCLM3, Hep3B, MHCC97H, SMCC7721) Immortalized liver cell lines (HL7702, HEK293T) Animal model	↓ cell proliferation	FAT10 promote CCN4 degradation to promote cancer progression	[113]
	Upregulated	Human hepatocellular carcinoma tissue (n = 80) Human hepatocellular carcinoma cell line (SMCC-7721)	CCN4 (siRNA) KD: ↓ cell invasion and migration		[80]
	Downregulated	Human hepatocellular carcinoma tissue (n = 80)			[84]
Lung cancer	Upregulated	Human lung cancer tissue (n = 60)			[73]
	Upregulated	C57BL/6-derived lung carcinoma cell line C57BL/6-derived melanoma cell line			[114]
	Upregulated	Human lung cancer cell lines (H1299, H460) Animal model	↓ cell motility, invasion, and lung metastasis	CCN4 decrease MMP1 expression and mediate integrin-αVβ5 and α1 dependent- Rho GTPase (Rac) inhibition	[37]
Oral cancer	Upregulated (28.57% cases)	Oral squamous cell carcinoma tissue (n = 95) Oral squamous cell carcinoma cell line (SCC-1483)	CCN4 (siRNA) KD: ↓ cell invasion and ↑ apoptosis		[79]
	Upregulated (24% cases)	Oral squamous cell carcinoma tissue (n = 204) Public database			[115]
	Upregulated	Oral squamous cell carcinoma serum (n = 62) Oral squamous cell carcinoma cell line (SCC4) Public database	↑ cell migration and EMT	CCN4 activates integrin αVβ3/FAK/ILK/Akt/Snail pathway and downregulates E-cadherin, miR-153-3p expression	[92]
	Upregulated	Oral squamous cell carcinoma tissue (n = 53) Oral squamous cell carcinoma cell lines (SCC4, CAL-27)	↑ cell migration, invasion, and wound healing CCN4 (shRNA) KD: ↓ migration	CCN4 increase ICAM-1 expression via αVβ3/ASK1, JNK/p38/c-Jun/AP-1 pathway	[93]
	Upregulated	Oral squamous cell carcinoma tissue (n = 47) Oral squamous cell carcinoma cell line (SCC4) Human endothelial cell line (HUVEC) Tumor xenograft model Public database	↑ angiogenesis	CCN4 increase VEGF-A expression via αVβ3/FAK/c-Src pathway transactivating EGFR/ERK/HIF-1α signaling	[94]
	Upregulated	Oral squamous cell carcinoma tissue (n = 60), Oral squamous cell carcinoma cell lines (SCC4, SAS) Human lymphatic endothelial cell line (LEC) Public database	↑ cell migration and tube formation	CCN4 increase VEGF-C and decreases miRNA-300 expression via αVβ3/ILK/Akt signaling pathway	[95]
Osteosarcoma	Upregulated	Human osteosarcoma tissue (n = 13) Human osteosarcoma cell lines (U2OS, HOS)	↑ cell migration and wound healing CCN4 (shRNA) KD: ↓ migration	CCN4 increase MMP2 and MMP9 expression via integrin αVβ3/Ras/Raf-1/MEK/ERK/NF-κB pathway	[32]
	–	Human osteosarcoma tissue (n = 20) Human osteosarcoma cell line (MG-63) Human endothelial progenitor cell line	↑ angiogenesis, cell migration and tube formation	CCN4 increases VEGF-A expression via integrin αVβ3/FAK/JNK/HIF-1α and decreases miR-381 expression	[116]
Ovarian cancer	Upregulated	Ovarian cancer tissue (n = 57) Ovarian cancer cell lines (IOSE80, SKOV3, CAOV4, CoC1) Tumor xenograft model	↑ cell proliferation, migration, invasion and EMT	CCN4 facilitate interaction between integrin αVβ3 and IGF1R	[74]
	Upregulated	Human ovarian cancer plasma (n = 20)			[117]
Prostate cancer	Upregulated	PC3-Luc luciferase expressing human prostate cancer cell line Transgenic adenocarcinoma mouse prostate model (TRAMP) in athymic immunocompromised mice	↑ cell migration, invasion, tumor growth and its spread to bones Anti-CCN4 antibody: ↓ tumor size, spread, and bone invasion		[118]
	-	Human prostate cancer cell line (LNCaP)	miR-29b-3p suppress CCN4 to facilitate mitochondrial apoptosis pathway	CCN4 inhibit Bcl-XL and activate caspase-3 and PARP	[119]
	Upregulated	Human prostate cancer tissues (n = 496) Public database	↑ CD8 + T cell infiltration and EMT gene signature ↑ inflammatory cytokine production (TNF-α, IFN-γ, IL-6)		[120]
	–	Human prostate cancer cell line (C4-2)	↑ cell adhesion, VCAM-1, and endothelin-1 expression	CCN4 promote adhesion via VCAM-1/ integrin α4β1 pathway	[121]
		Human prostate fibroblast cell lines (HPrF, WPMY-1) Human prostate carcinoma cell line (PC-3)	↑ cell proliferation, migration, invasion, tumor growth and prostate fibroblasts contraction CCN4 (shRNA) KD: ↓ cell proliferation and contraction	CCN4 isoform upregulate N-cadherin, SNAIL, slug, and vimentin and inhibit NDRG1	[122]
	Upregulated	Human prostate cancer cell lines (LNCaP, DU145 and PC-3, PZ-HPV-7) Osteoblast-like cell line (MG-63)	↑ tumor growth, EMT and metastasis	CCN4 elevated N-cadherin and Twist expression and reduced E-cadherin expression	[123]
	–	Human prostate cancer cell lines (WPMY-1, PC3, LNCaP, DU145, 22Rv1)	↑ cell proliferation, migration, and invasion	miR-145-3p and circular RNA, circ_KATNAL1 decrease CCN4 expression and effect CCN4 increase MMP2, MMP9 and inhibit caspase 3/8/9 and PARP expression	[124]
		Human prostate cancer cell lines (PC3, DU145)	↑ cell migration, VCAM-1 expression, and ↓miR-126	CCN4 increase phosphorylation of FAK and p38 via integrin αVβ1	[91]
Skin cancer/Melanoma	Upregulated	Mouse melanoma cell lines (B16F0, B16F10, YUMM1.1, YUMM1.7) Human metastatic melanoma cell lines (RPMI-7951, SK-MEL-3. SH-4) Animal model (CCN4-CRISPR KO mice and NSG-xenograft tumor model)	CCN4 (CRISPR) KO: ↑ cell proliferation and ↓ invasion, migration, wound healing and EMT	CCN4 increases phosphorylation of Akt and MEK/ERK to induce EMT	[125]
	–	Mouse melanoma cell lines (B16F0, B16F10) Human metastatic melanoma cell lines (RPMI-7951, SH-4, SK-MEL-3, SK-MEL-24) Mouse fibroblast cell line (NIH3T3) Animal model (CCN4-CRISPR/Cas9 knockout) Public database	↑ cell migration, invasion and EMT CCN4 (CRISPR) KO: ↑ cell proliferation and ↓ cell migration, invasion, wound healing	CCN4 knockout increased E-cadherin, Zeb1 and decreased Snail2, Zeb2, N-cadherin, vimentin, and fibronectin	[126]
	–	Mouse melanoma cell lines (B16F0, YUMM1.7) Animal model (NSG-xenograft tumor model) Public databases	CCN4 (CRISPR) KO: ↓ tumor growth and ↑ CD8^+^ T cells, CD45^+^ leukocytes and ↓ myeloid-derived suppressor cells tumor infiltration (↑ immunosuppression)	CCN4 enhance CCL2 and CXCL1 release from tumor and decrease IFN-γ release from CD8^+^ T cells	[81]
	Downregulated	Human melanoma tissue (n = 3) Human melanoma cell lines (1205Lu, Sbcl2, WM35, WM278, WM3248, WM3899) Human primary dermal fibroblasts Tumor xenograft model	↓ cell growth CCN4 (shRNA) KD: ↑ cell proliferation and ↓ apoptosis		[85]
	–	Mouse melanoma cell line (B16F0) Animal model (NSG-xenograft tumor model) Public database	CCN4 (CRISPR) KD: ↓ tumor growth, ↑ CD45^+^ lymphocytes, particularly natural killer cells		[127]

↑, promotes; ↓, KD: knockdown; KO: knockout; inhibition; Akt: protein kinase B; AP-1: activator protein-1; ASK1: apoptosis signal regulating kinase 1; Bcl-XL: B-cell lymphoma-extra-large; CCAT-2: Colon cancer associated transcript-2; CCL: chemokine C–C motif ligand; CD: cluster of differentiation; c-myc: cellular myelocytomatosis oncogene; c-Src: cellular Src tyrosine kinase; CXCL1: chemokine C-X-C motif ligand 1; EGFR: epidermal growth factor receptor; EMT: epithelial mesenchymal transition; ERK: extracellular signal-regulated kinase; FAK: Focal adhesion kinase; FAT10: F locus adjacent transcript 10; GLUT1: glucose transporter 1; HIF-1α: hypoxia inducible factor 1 alpha; ICAM-1: intracellular adhesion kinase 1;IFN- γ: interferon gamma; IGF1R: insulin growth factor-1 receptor; IL-12: interleukin 12; ILK: integrin linked kinase; JNK: c-Jun N-terminal kinase; MEK: mitogen-activated protein kinase kinase; miR: microRNA; MMP: matrix metalloprotease; mTOR: mechanistic target of rapamycin;NDRG1: N-myc downstream-regulated gene 1; NF-κB: nuclear factor kappa B; p38: mitogen-activated protein kinase; p53: tumor suppressor protein; PARP: poly ADP-ribose polymerase; PI3K: phosphoinositol 3-kinase; RNA AFAP1-AS1: long noncoding RNA actin filament associated protein 1 antisense RNA1; Runx2: Runt-related transcription factor 2; Slug: snail family transcriptional repressor 2; SNAIL: Zinc finger protein SNAI1; TEAD1: TEA domain transcription factor 1; TGF-β: transforming growth factor beta; Twist: twist family bHLH transcription factor; VCAM-1: Vascular cell adhesion protein 1; VEGF: vascular endothelial growth factor; YAP1: Yes-associated protein 1; Zeb: Zinc finger E-box-binding homeobox 1; γ-H2AX: repress histone2A family member X

Tumor microenvironment is composed of cancer cells, stromal cells, such as immune cells, fibroblasts, endothelial cells in conjunction with ECM, all of which are constantly communicating and influencing each other via cytokines, chemokines and inflammatory signaling molecules [86]. Although numerous cell-based and animal experimental models have been utilized to study the effect of CCN4 in cancer, majority of the studies are conducted in an isolated single cancer cell line model, which fails to capture the intricate crosstalk amongst different cell types within the tumor microenvironment. Given that CCN4 is a secreted protein, better understanding of both autocrine and paracrine effects is crucial to decipher the functional disparity and clinical significance of CCN4 to develop novel pharmacological interventions targeting CCN4 in cancer [87]. Furthermore, mechanistic data is derived from either loss-of function approach, including shRNA, siRNA or CRISPR mediated transcriptional repression or protein overexpression approach using viral vector. Every experimental approach has its own caveats, here both approaches may fail to capture the true function of endogenous CCN4 due to the off-target effects and lack of specificity leading to partial loss-of function or by attaining supraphysiological protein levels [88, 89]. Some studies also utilize recombinant CCN4 protein to understand its biology, which has its own drawbacks. Besides the time-consuming and costly process, the multi-modular structure of CCN4 is susceptible to proteolytic cleavage generating functionally active or in-active truncated variants. Additionally, species to species variations in post-translational modification patterns could also influence the biological activity of the protein [90]. Hence, caution must be exercised while interpreting the functional consequences of full-length CCN4 protein.

Molecular pathways governing CCN4 mediated cellular oncogenic responses remains undiscovered, due to the lack of one CCN-specific cognate receptor. However, within the limited available literature, integrins have been identified as the major contributors in driving CCN4-dependent biological responses. Researchers have shown that CCN4 binds and interacts with integrins, such as, αVβ1, αVβ3, αVβ5, α4β1, α5β1 and α6β1 in myriad cancerous conditions to drive cell proliferation, migration, invasion etc. making them one of the most extensively documented functionally significant receptors for CCN4 till date [32, 33, 37, 74, 87, 91–95]. In addition, CCN4 polymorphism directly influences the risk of developing tumor [96, 97], disease progression [98], response to chemotherapy toxicity [99] and CCN4 expression via epigenetic modulation such as DNA methylation [100].

### CCN4 in fibrosis

Fibroblasts are highly plastic cells with mesenchymal origin found throughout the body to provide structural integrity and basic framework for cells and tissues. Fibroblasts are elongated stellate shaped cells that are most commonly present in the stroma. They are responsible for maintaining, synthesizing, and organizing ECM proteins, such as collagen, fibronectin (FN1), laminins etc. and therefore, play a key role in wound healing and tissue repair [128]. Mechanical or chemical stimuli from the site of injury can activate the otherwise quiescent tissue resident fibroblasts and initiate their transformation into myofibroblasts. Under physiological homeostasis, once the wound healing and repair has been completed, myofibroblasts undergo apoptosis to prevent excessive ECM deposition. However, chronic persistent injury and insult, can dysregulate and disrupt the body’s natural restorative process, leading to excessive ECM deposition, tissue scarring and architectural remodeling, loss of tissue elasticity and function, resulting in fibrosis [129]. Fibrosis is the most common pathological outcome in chronic inflammatory conditions related to lungs (Idiopathic pulmonary fibrosis; IPF), skin (scleroderma), kidney diseases, liver, and heart (cardiac fibrosis) [130].

Fibrosis is a chronic, highly progressive, and irreversible condition that is the leading cause of organ dysfunction and death. Although, two FDA (Food and Drug Administration) approved drugs, Nintedanib and Pirfenidone provide symptomatic relief in IPF, there are currently no drugs that address the underlying cause to cure fibrosis [131]. Emerging evidence indicates that CCN4 protein is pro-fibrotic in nature and modulates fibroblast proliferation. CCN4 is highly upregulated in both pre-clinical bleomycin model of pulmonary fibrosis, paraquat induced model [132, 133] and in clinical IPF patients compared to non-diseased control. Furthermore, Klee and colleagues demonstrated that CCN4 is downstream of TGF-β and TNF-α, which are the master regulators of fibrosis and inflammation. CCN4 promotes the proliferation of human lung fibroblast in an IL-6 dependent manner as siRNA mediated knockdown or antibody-mediated neutralization of CCN4 abrogates the effect [63]. The CCN4 upregulation could be partly due to the downstream effects of TGF-β in conjunction with the downregulation of microRNAs (miRNAs), particularly miR-92a, which has been shown to modulate CCN4 expression. miR-92a expression is inversely corelated with CCN4 expression in IPF patient lung specimens [134]. In addition, miR-101 and miR-181a-5p regulate CCN4 expression in cystic fibrosis [135]. Airway epithelial cells not only act as the first line of defense against environmental threats but also serve as a dynamic junction to relay the extracellular signal to other immune cells that underlay smooth muscle cells, fibroblasts and myofibroblasts. Chronic epithelial insult and dysfunction have been attributed to the pathogenesis of asthma and IPF [136–138]. Heise and colleagues demonstrated that mechanical stress and stretch can induce CCN4 expression in primary mouse type II alveolar epithelial cells (AT-II cells) and drive epithelial to mesenchymal transition (EMT). Further, CCN4 neutralizing antibody significantly abrogated stretch induced EMT, emphasizing the critical role of CCN4 in EMT [139]. Similar findings were validated by another group, where treatment with recombinant CCN4 (1 µg/ml) promoted cell proliferation and EMT in primary mouse AT-II cells. In addition, stimulation with CCN4 (1 µg/ml) for 6 to 12 h upregulates fibrotic genes, such as Col1A1, Col1A2 and FN1 in mouse and human fibroblasts and the effects were attenuated in the presence of a CCN4 neutralizing antibody in the bleomycin model [60]. CCN4 mediated cell adhesion in airway epithelial cells (A549) is partly mediated by integrins, as αVβ5, αVβ3 or αVβ1 neutralizing antibodies partially blocked the effect [140]. Irradiation has been shown to upregulate CCN4 expression in human lung fibroblasts with implications in radiation-induced lung injury in cancer patients [141]. Nintedanib, a small molecule receptor tyrosine kinase inhibitor, approved for IPF has also been shown to regulate Wnt/β-catenin pathway and prevent myofibroblast activation by inhibiting CCN4 in mouse lung myofibroblast cell line, Mlg [142]. Furthermore, secreted CCN4 was significantly decreased upon treatment with Nintedanib (1 µM) in ex-vivo 3D-human lung tissue. While CCN4 levels remained unaffected upon treatment with Pirfenidone (500 µM) detected by ELISA (Enzyme-linked immunosorbent assay) [143]. However, in another study, both Nintedanib (0.3 µM) and Pirfenidone (1 mM) reduced in precision cut rat-lung slices [144]. CCN4 can also facilitate inflammatory response in fibrosis. In addition to IL6 and CCL2 production [63, 145]. CCN4 also mediates the release of the proinflammatory cytokine TNF-α from macrophages (RAW264.7) in an integrin αVβ3 dependent manner and regulates TLR4 signaling in an acute lung injury pre-clinical model [146].

Apart from pulmonary fibrosis, CCN4 has also been involved in liver fibrosis and inhibition of CCN4 can reverse liver fibrosis [147–149]. Stimulation with pro-fibrotic/ pleotropic cytokines, such as TGF-β and TNF-α increased CCN4 induction in-vitro in hepatic stellate cell lines (LX-2 and HSC-T6/ HSC). In addition, recombinant CCN4 drives LX-2 cell proliferation in a dose-dependent manner. CCN4 protein expression was also significantly upregulated in-vivo in carbon tetrachloride (CCl_4_)-induced liver fibrosis model [148] and CCN4 antibody significantly decreased pro-fibrotic protein expression (collagen, α-smooth muscle actin (αSMA), TGF-β1), reduced liver necrosis, NF-κB activation and pro-inflammatory cytokine production, such as IL-6, CCL-2 and TNF-α [149]. Huang and colleagues utilized RNA sequencing analysis to identify a set of differentially expressed genes in ex-vivo precision-cut lung tissue slice to design a robust biomarker panel to assess antifibrotic effects of various interventions. CCN4 was amongst other genes and secretory proteins in the panel and was used as a reliable end point parameter to evaluate the efficacy and anti-fibrotic activity of the compounds [144]. Interestingly, as the molecular mechanisms driving the initiation and progression of fibrosis remain poorly understood, recent study identified a novel pathway dissecting the role of CCN4 in the progression of liver fibrosis and not initiation of the disease as CCN4 knockout animals were protected against liver-fibrosis progression in pre-clinical CCl_4_-liver fibrosis and choline-deficient, L-amino-acid-defined, along with the high-fat diet (CDA-HFD)-induced NASH models. Furthermore, functional analysis confirmed that CCN4 mediated fibrogenesis and myofibroblast motility is partly driven by integrin (αV, α11) dependent myocardin-related transcription factor (MRTF) activation, that drives MRTF-downstream cytoskeletal gene targets, such as αSMA, myosin light chain 9, filamin A, etc., in primary HSCs. Although the precise mechanism of fibrosis remains elusive, evidence from current literature indicate that CCN4 could serve as a potential therapeutic target for the treatment of liver fibrosis and small or large molecule therapeutic modalities that inhibit CCN4 can elicit protective effects in liver injury and fibrosis.

CCN4 has a crucial role in skin biology, wound healing, and repair. CCN4 protein expression was upregulated 4–7 days post cutaneous wounding and facilitated wound healing as the extent of wound closure was significantly delayed in CCN4-knockout mice due to the downregulation of ECM proteins, such as Col1A1 and FN1 [150]. Immunohistochemistry analysis of the incision reveals that CCN4 is also abundantly expressed in inflammatory cells, such as neutrophils. CCN4 is crucial for wound healing as it drives proliferation and migration of both human and mouse dermal fibroblasts through integrin α5β1 as selective siRNA mediated CCN4-knockdown resulted in the loss of function. Furthermore, stimulation with 100 ng/ml CCN4 induced activation and phosphorylation of ERK and c-Jun N-terminal kinase (JNK), crucial for cell proliferation, an effect which was blocked in the presence of selective small molecule MAPK inhibitor, PD98059 and αVβ1-antibody [150]. CCN4 not only binds with integrins but can also interact with cell surface small-leucine rich proteoglycans such as, decorin and biglycan on human dermal fibroblasts [25], although the downstream mechanistic pathway engagement from the latter remains unknown.

Besides the role of CCN4 in lung, liver, and skin fibrosis, CCN4 has also been shown to be implicated in cardiac remodeling and fibrosis associated with cardiomyopathies. Similar to what others have shown in diverse pathological conditions, CCN4 is substantially upregulated post-myocardial infarction (MI) and ischemic injury [64, 65, 151, 152]. CCN4 modulates cardiac remodeling by positively influencing cardiomyocyte hypertrophy in an Akt-dependent manner and stimulation with recombinant CCN4 induces cardiac fibroblast proliferation and enhances ECM protein deposition, particularly collagen [65]. As previously mentioned, pro-inflammatory cytokines and chemokines are closely intertwined with CCN4 biology, and both have been shown to positively regulate each other. Along the same lines, stimulation with either TNF-α and/or IL-1β significantly induced CCN4 protein expression both in left-ventricular myocardium post-MI in-vivo and in rat cardiac myocytes in-vitro [65]. These findings were further strengthened in another study, where TNF- α induced CCN4 upregulation was shown to be dependent on ERK1/2 mediated CREB phosphorylation at Ser133 as pretreatment with small molecule inhibitors such as PD98059 (ERK1/2) and U0126 (MEK) failed to induce CREB-phosphorylation [64]. As TNF-α is known to activate a vast array of downstream signaling molecules, the authors also ruled out the involvement of JNK- and NF-κB activation for CCN4 upregulation and concluded that TNF-α mediated responses were strictly dependent on MEK1-ERK1/2-CREB signaling in cardiac fibroblasts [64]. In addition to CCN4, biglycan, the potential binding partner of CCN4 is also upregulated in cardiac fibroblasts up to threefold post-MI in-vivo suggesting intracellular CCN4 signal amplification [65].

Renal fibrosis is one of the most common pathological hallmarks in chronic kidney diseases (CKD). A recent study found that CCN4 levels were highly upregulated in preclinical unilateral ureteral obstruction (UUO) renal fibrosis model in animals and clinically in serum and kidney tissue biopsy samples from CKD patients [153–155]. Mechanistically, antibody mediated neutralization or siRNA mediation knockdown of CCN4 provides protection against renal fibrosis by attenuating fibrotic markers, such as Collagen, FN1 and αSMA deposition both in tubular epithelial cells (NRK52E cell line) and in-vivo in mouse models. Interestingly, the role of autophagy in fibrosis remains controversial as there are opposing results on whether it promotes or inhibits fibrogenesis. However, previous investigations have reported enhanced autophagic markers in proximal tubular cells, pharmacological inhibition of which reversed renal fibrosis. Similarly, CCN4 inhibition significantly reduced autophagy in UUO renal fibrosis model, suggesting that CCN4 also exercise control over pathways governing programmed cell death, modulating the development of renal fibrosis [156]. Serum CCN4 levels were also found to be elevated in vast array of CKDs (chronic kidney disease), such as diabetic nephropathy, IgA nephropathy and primary focal segmental glomerular sclerosis [153, 157]. CCN4 has also been implicated to drive migration, invasion and EMT in primary renal tubular epithelial cells in uremia associated with end-stage renal failure [53]. In addition, the growing body of literature on non-coding RNAs, particularly miRNA and circular RNA (circRNA) and its prominent role in the pathophysiology of a wide array of disease highlights them as potent gene regulators. Regarding CCN4 gene regulators, miR-92a, miR-101 and miR-181a-5p have been identified to inversely modulate CCN4 expression in lungs. A recent study discovered two novel non-coding RNAs that target CCN4 to modulate renal fibrosis. The results demonstrate that circRNA-33702 is overexpressed in UUU-renal fibrosis models and possess profibrotic role by aggravating collagen and FN1 expression [158]. Conversely, miR-29b-3p negatively modulates CCN4 expression, in conjunction with other ECM proteins in mouse proximal tubule cell line (BUMPT cells). Since circRNA-33702 and miR-29b-3p have opposing effects on CCN4 expression and colocalization, luciferase analysis revealed that circRNA-33702 directly binds miR-29b-3p to upregulate CCN4 expression and consequently promote renal fibrosis [158]. The anti-fibrotic effects of miR-29b-3p on cardiac and liver fibrosis have also been demonstrated by others [159–162]. Apart from miR-29b, long non-coding RNA, Gm12840 and miR-677-5p also target CCN4/Akt signaling pathway to modulate fibroblast activation in ischemia–reperfusion induced renal fibrosis [163]. Overall, non-coding RNAs possess exciting potential as novel therapeutic targets for the treatment of fibrosis, however more investigation in this area is required to comprehensively understand the crosstalk amongst non-coding RNAs in a tissue-specific context to target CCN4.

In addition, all the CCN family members are functionally interconnected with a high degree of crosstalk by compensatory or opposing mechanisms. Emerging evidence points towards the anti-fibrotic effects of CCN3 protein and one of the possible anti-fibrotic mechanisms involves downregulation of profibrotic CCN4 protein. Overexpression of CCN3 in the skin fibroblast cell line NIH3T3 significantly downregulated CCN4 expression and hence conferred protection against fibrosis, although the exact mechanisms by which CCN3 modulates CCN4 remains unknown. It is speculated that due to its presence in the nucleus, CCN3 may behave as a transcription factor and can directly inhibit CCN4 gene transcription. Another mechanistic explanation could be due to direct sequestration of CCN4 by protein–protein interaction, preventing the initiation of CCN4 mediated pro-fibrotic pathway [164]. Similar findings were also reported by others where CCN3 was identified as an endogenous inhibitor of pro-fibrotic CCN family members. A deeper understanding of the interactome of CCN family members is required for utilizing the antagonistic approach to develop anti-fibrotic therapeutics [165, 166]. Taken together, these results show that CCN4 is highly upregulated in fibrotic tissues, such as lungs, heart, liver and skin and can directly influence fibrogenesis, partly by integrin dependent mechanisms to promote fibroblast proliferation and migration, suggesting that CCN4 can not only serve as a diagnostic biomarker but also be exploited as a novel therapeutic target for the treatment of fibrosis. In addition, targeting CCN4 may eliminate the need for multiple tissue specific therapies in multi-organ fibrosis, given its role in the fibrogenesis of major organs, such as lung, liver, heart, kidney, and skin which encompasses a majority of all the fibrosis cases.

### CCN4 in obesity and diabetes

Obesity is defined as excessive accumulation of fat in the adipose tissue throughout the body due to imbalance in the energy intake and expenditure, leading to various cardiovascular and metabolic disorders. Numerous environmental, genetic and lifestyle related factors also contribute to the increased body mass index (BMI) and weight gain [167]. Previously considered to be inactive, adipose tissue is a highly dynamic metabolically active endocrine organ, that produces a wide variety of cell-signaling molecules, namely adipocytokines or adipokines such as leptin, adiponectin, resistin, TNF-α, and IL-6, amongst others [168]. These adipokines are crucial for biochemical and metabolic homeostasis, however increased adiposity mediated adipokine dysregulation is the major culprit involved in the pathogenesis of metabolic syndrome, such as insulin resistance, diabetes mellitus, atherosclerosis, etc. [169]. CCN4 has recently been identified as a novel adipokine in humans, adding a unique functional aspect to its diverse biological repertoire [170]. Interestingly, recent studies have shown that CCN4 is also expressed and secreted by human adipocytes endowing it with the title of novel adipokine. Amongst other CCN protein members, CCN3 is also a fairly recently discovered adipokine linked to obesity [171]. Discovery of CCN4 and CCN3 as novel metabolic regulators opens new avenues for the treatment and management of obesity and associated co-morbidities.

Over the last decade, emerging evidence directly correlates systemic CCN4 levels with obesity, inflammation, and insulin-resistance. Large human cohort studies with obese and/or glucose tolerant patients revealed that circulating CCN4 is positively correlated with percent fat mass, leptin, triglyceride levels, adiposity, and BMI [172, 173]. Another study shows that serum CCN4 levels and CCN4 mRNA expression in visceral adipose tissue were significantly higher in obese men compared to non-obese men, independent of their glycemic status [174]. CCN4 also leads to insulin resistance by impairing insulin signaling in hepatocytes and primary human skeletal muscle cells. In a dose-dependent manner, CCN4 significantly abrogated insulin-mediated phosphorylation of insulin receptor (IRβ)-Tyr1150/1151, along with decreased Akt-Ser473/Thr308, GSK3β-Ser9 phosphorylation at the lowest dose of 0.1 µg/l in both human skeletal muscle cells and murine hepatocyte cell line AML12. Insulin receptor substrate 1 (IRS1) is a key cytoplasmic adaptor protein crucial for signal transmission downstream of the receptor and treatment with 0.1 µg/l and 1 µg/l CCN4 decreased IRS1 protein expression by 50%, suggesting the direct inhibitory effect of CCN4 on insulin cascade in human skeletal muscle cells [174]. Preincubation with 0.1 µg/l and 1 µg/l of CCN4 for 24 h significantly abrogated insulin-dependent glycogen synthesis in human primary myotubes [174]. To further validate the mechanism, Woo et al. demonstrated the effect of CCN4 knockdown on insulin resistance and glucose in skeletal muscle cells of HFD-mice [175]. CCN4 knockdown significantly abrogated the inhibitory effects on insulin signaling by restoring Akt and IRS1 phosphorylation. Mechanistically, the authors also showed that CCN4 mediated insulin resistance and inflammation in murine skeletal muscle cells (C2C12 cells) and hepatocytes respectively is driven via Toll-like receptor-4 (TLR4) as TLR4 knockdown significantly abrogated CCN4-mediated JNK phosphorylation, NF-κB translocation, insulin resistance and triglyceride accumulation in hepatocytes and C2C12 cells [175]. siRNA mediated knockdown of NF-κB and JNK prevents CCN4-mediated insulin resistance, highlighting a novel mechanism for CCN4-driven impaired insulin sensitivity. In addition, siRNA-mediated CCN4 knockdown significantly ameliorates hepatic steatosis, lipogenesis and insulin resistance in HFD-fed mice suggesting that CCN4 requires TLR4 activation to drive inflammation and insulin resistance [175].

Contrary to the previously described inhibitory effect of CCN4 on insulin signaling, another study reports that it drives insulin-producing pancreatic beta (β)-cell proliferation via Akt modulation in both mouse and human cells [176]. Cell proliferation markers such as antigen kiel 67 (Ki67) and phospho-histone H3 (pHH3) were significantly reduced in CCN4 knockout mice (CCN^−/−^) as compared to wild-type (CCN^+/+^). Further, CCN^−/−^ mice treated with recombinant CCN4 exhibited twofold higher β-cell proliferation as compared to saline. Adenovirus-mediated systemic overexpression of CCN4 in streptozotocin-induced diabetes model, significantly increased plasma insulin levels by augmenting total β-cell mass and insulin positive area, however failed to reverse hyperglycemia [176]. CCN4 has also been implicated in the development and regeneration of pancreas [177, 178]. More recently, researchers have also identified that CCN4 is highly expressed upon treatment with high concentration of glucose (30 mM) in human kidney proximal tubular cells and in renal tissue of streptozotocin-induced diabetic nephropathy (DN) mouse model [179]. Functionally, CCN4 overexpression drives cell proliferation, migration, EMT and fibrosis and these effects were partially rescued via silencing N6-adenosine methyltransferase (METTL3), which decreases DN development by decreasing CCN4 expression in-vitro [179]. Given the current body of conflicting literature on the effect of CCN4 on glucose homeostasis and rising rate of metabolic disorders, it is extremely important to decipher the functional and mechanistic consequences of CCN4 due to its profound physiological relevance.

Two independent studies have also shown that CCN4 serum level in pregnant women with gestational diabetes mellitus (GDM) is significantly higher as compared to healthy non-GDM pregnant women [180, 181]. In addition, circulating CCN4 level in obese pregnant women with GDM is positively correlated with numerous clinical metabolic parameters such as systolic blood pressure, fasting blood glucose and aspartate aminotransferase (AST), highlighting the crucial role of CCN4 in the pathophysiology of GDM [180]. Overall, CCN4 can serve as a strong independent risk predictor and diagnostic marker and possess immense therapeutic potential in maternal-neonatal health and obstetric research. However, maternal, and neonatal safety drug assessment becomes crucial to assess the impact of possible CCN4 interventions on the fetal growth and development as CCN4 is expressed in osteoblasts and their progenitor cells during skeletogenic processes in embryonic development [182].

Obesity is also characterized as a chronic low-grade systemic inflammation due to the proinflammatory cytokine release from adipocytes and macrophages [183]. Studies show that macrophage infiltration and accumulation in adipose tissue was significantly higher in obese HFD mice as compared to normal mice [184]. Furthermore, phenotypical polarization of the infiltrated macrophages was observed in obese individuals with predominant pro-inflammatory M1 macrophages as compared to lean individuals with more anti-inflammatory M2 macrophages. The increased M1 population in obese adipose tissue overexpresses pro-inflammatory genes such as IL-6 and TNFα and lower anti-inflammatory cytokines such as IL-10, contributing to the persistent low-grade systemic inflammation and insulin resistance [184, 185]. In addition, CCN4 protein expression is upregulated in visceral and subcutaneous adipose tissue in glucose-tolerant patients and is positively correlated with the markers of obesity, inflammation, and insulin resistance [170, 172, 174]. Serum CCN4 levels were also significantly elevated in obese children and adolescents with direct positive correlation to IL-18, adiponectin, and leptin [61]. Interestingly, these effects were completely reversed upon weight loss as adipose tissue CCN4 expression was significantly decreased after weight reduction, suggesting that adipocytes are the major source of circulating CCN4 [170]. These findings were further validated in another single-center randomized trial with breast cancer survivor females, where a 12-week exercise regime significantly decreased waist circumference and body fat composition accompanied by reduced serum β-catenin and CCN4 levels [186]. Furthermore, studies have shown that stimulation of macrophages with CCN4 can significantly increase pro-inflammatory cytokines such as IL-6, TNF-α and IL1B at both mRNA and protein level [170, 187]. However, CCN4 stimulation had no significant pro-inflammatory effect on adipocytes suggesting that adipocyte-derived CCN4 does not elicit autocrine-response but rather have paracrine inflammatory effects on nearby macrophages. Additionally, stimulation of macrophages with CCN4 significantly increased the expression of pro-inflammatory M1 specific markers, such as CCR7 and COX2, whereas the expression of anti-inflammatory M2 specific markers such as CD36, CD163, MRC1 and COX1 were either markedly decreased or remained unchanged. This suggests that ‘M2 to M1’ phonotypical switch is driven at least in part due to adipocyte derived CCN4, along with other unknown mechanisms [170]. CCN4 alone does not initiate the release of inflammatory cytokines from adipocytes, however CCN4 imparts protective effects on LPS-treated adipocytes (3T3-L1) by preventing cell apoptosis and injury [188].

Murahovschi and colleagues have reported increased CCN4 expression and release during adipocyte differentiation, however no effect of CCN4 on adipocyte differentiation [170]. Yet another conflicting report suggests that CCN4 expression significantly decreases during adipocyte differentiation from preadipocytes to mature adipocytes and negatively regulates adipogenesis by physically interacting and redirecting transcriptional factor peroxisome proliferator-activated receptor gamma (PPARγ) to proteasomal degradation, which serves as a master regulator of adipocyte differentiation [189]. Potentially CCN4 does not promote new adipocyte formation, however, it can maintain and protect the pre-existing adipocytes that contribute heavily to the circulating CCN4 levels. CCN4 positively self-regulates itself by protecting the source, i.e., adipocytes, which can further aggravate systemic CCN4 levels, worsening the condition.

Increased adiposity and overexpressed CCN4 in adipocytes of obese individuals significantly contribute to the pro-inflammatory cytokine release by stimulating adipose tissue resident macrophages in a paracrine fashion and drives macrophage polarization with can further worsen the condition. Similarly, supraphysiological serum CCN4 levels also lead to insulin resistance by impairing insulin signaling. Taken together, all the evidence indicates that CCN4 is a central player and key contributor towards aggravation and perpetuation of the inflammatory response and insulin desensitization in obesity. Targeting CCN4 could have great therapeutic potential for metabolic disorders that would benefit numerous patients across the globe.

### CCN4 in musculoskeletal system-osteoarthritis (OA) and rheumatoid arthritis (RA)

As per CDC, Arthritis is the leading cause of disability affecting nearly 1 out of 4 adults in the US. Arthritis means ‘disease of the joints’ and is usually characterized by chronic inflammation, pain, stiffness, loss of mobility and function due to progressive damage to the joint, bone and cartilage [190]. OA and RA are the most prevalent joint decaying diseases, with diverse etiologies but overlapping clinical hallmarks [191]. While there is no cure for debilitating OA and RA, treatment paradigms focus on symptomatic relief using a combination of pain management and physical therapy. A better functional and mechanistic understanding of the disease will assist in the discovery of novel diagnostic and therapeutic biomarkers for developing novel treatment strategies [190]. Given the diverse pharmacological effects of CCN4 in the human body, emerging evidence indicates the involvement of the Wnt-pathway in joint diseases.

The significance of CCN4 in the pathophysiology of OA and RA has been abundantly demonstrated by numerous researchers over the last decades. Substantial evidence implicates deleterious effects of CCN4 in the development of musculoskeletal disorders. Differential gene expression and transcriptomics analysis revealed significantly higher CCN4 expression in the cartilage of OA patients as compared to healthy controls [52, 100, 192–196]. Spatial expression profile of CCN4 reveals moderate to weak expression in the superficial layer, matrix and synovial perivascular cells of the knee and hip of RA and OA patients [192]. Another study revealed notable CCN4 upregulation in both synovium and cartilage specimens from OA-human patients and collagenase-induced OA mouse model [52, 195]. CCN4 augmentation has been shown to stimulate chondrocytes, synovial cells, and macrophages, to induce the expression of matrix-degrading proteolytic enzymes such as MMPs that impart deleterious effects on the joint tissue of OA and RA patients. Adenovirus-mediated CCN4 overexpression in knee joints of naïve mice significantly damaged cartilage by inducing MMP13, MMP9, ADAMTS-4 and ADAMTS-5 in synovium and cartilage, exacerbating the condition [52]. In addition, treatment with recombinant human CCN4 increases MMP1, MMP2, MMP3, MMP9 and MMP13 mRNA expression in human OA synovial specimens [197]. Interestingly, CCN4 expression is directly corelated with OA severity and was densely expressed in the most damaged and degraded areas of the joint, confirming its detrimental effects, and highlighting the key role of CCN4 in OA and RA progression [198]. These findings were also confirmed in another study that demonstrated a direct role of CCN4 in the pathogenesis of OA utilizing CCN4-knockdown approach in three different experimental models of OA, that is, intra-articular collagenase induced (CIOA), anterior cruciate ligament transection (ACLT) and destabilization of the medial meniscus (DMM) model. Cartilage degradation was significantly decreased in CCN4^−^/^−^ mice in all three OA-models compared to WT (wild type). These effects were attributed to decreased expression of protease, MMP3, MMP9, ADAMTS-4 and ADAMTS-5 in the synovium of CCN4^−^/^−^ mice, suggesting CCN4 is one of the key culprits in OA-pathogenesis, [197]. Interestingly, miR-128-3p expression is notably decreased in human OA tissue, and overexpression of miR-128-3p significantly decreased CCN4 expression. Furthermore, CCN4 mediates chondrocyte apoptosis, inflammation, and ECM degradation via PI3K/Akt/ NF-κB pathway, an effect that was inhibited by miR-128-3p providing protection against the harmful effects of CCN4, emerging as a novel therapeutic target for OA [196]. Besides low miR-128-3p expression, elevated levels of TGF-β in OA drive CCN4 expression in chondrocytes [199]. CCN4 can also contribute to OA pathology by skewing TGF-β signaling in chondrocytes from protective/ non-hypertrophic ALK-5/Smad 2/3 pathway towards damaging/ hypertrophic ALK-1/ Smad 1/5/8 pathway [200].

As previously described CCN4 binds to certain integrins, predominantly αVβ5, αVβ3 and αVβ1 to mediate its functional effects in fibrosis and cancer. Similarly, researchers have also shown that CCN4 engages integrins expressed on chondrocytes and synovial fibroblasts in OA. Contrary to the previously reported degenerative effects of CCN4 on chondrocyte matrix, another study shows that CCN4 displays a protective effect on primary human OA articular chondrocytes by inhibiting senescence and apoptosis. This effect was blocked in the presence of either αVβ3 antibody or a potent small molecule PI3K inhibitor (LY294002), suggesting that CCN4 mediated protective effects are αVβ3/PI3K/Akt dependent [198]. In another study, pretreatment with αVβ5 integrin blocking antibody, but not αVβ3 or α5β1 significantly reduces CCN4 induced concentration and time dependent increase in IL-6 production in OA synovial fibroblasts. Furthermore, CCN4-dependent IL-6 production was also obliterated in the presence of PI3K (Wortmannin and LY294002), Akt (Akti) and NF-κB (TPCK and PCTC) inhibitors suggesting that CCN4 drives pro-inflammatory cytokine IL-6 release via αVβ5/PI3K/Akt/NF-κB pathway in OA [201]. Together with CCN4, the expression of integrin αV and α5 subunit was significantly higher in human OA cartilage as compared to controls and assists CCN4-dependent chondrocyte differentiation [199]. Chondrocytes are terminally differentiated cells, possessing poor self-restoring capacity leading to longer recovery time after the avascular cartilage injury. Chondrocyte dedifferentiation causes multiple phenotypical changes, that accelerates hypertrophy, matrix calcification, degradation, and fibrosis. Dedifferentiated chondrocyte markers were notably upregulated in OA cartilage, suggesting that in conjunction with other mediators, CCN4 also promotes the destructive dedifferentiation process, aggravating disease progression [202]. Contrary to the general consensus, CCN4 also promotes chondrocyte proliferation, independent of integrins [199]. Treatment with CCN4 have been shown to also drive primary human OA chondrocyte migration, however the migratory chondrocytes were not phenotypically characterized to conclude whether they are non-differentiated which could facilitate repair or dedifferentiated which can cause damage [203].

Stimulation of human-osteoblast-like cells with recombinant human CCN4 dose-dependently increases mitogenic activity assessed by BrdU incorporation and osteoblastic differentiation measured by alkaline phosphatase activity [49]. Another study reported positive influence of CCN4 on bone formation. CCN4 overexpression both in-vivo and in-vitro in osteogenic hBMSC drives osteogenesis, increased bone volume and thickness by increasing bone morphogenic protein 2 (BMP-2) expression and activity in an α5β1 integrin dependent manner [204]. Furthermore, stimulation of mesenchymal stem cells (MSC) with recombinant CCN4 stimulates proliferation in a dose-dependent manner via BMP-3 induction, as CCN4-siRNA mediated knockdown significantly reduced the mitogenic effects of BMP-3 [205]. CCN4 also promotes recruitment, adhesion, and migration of monocytes by dose-dependently increasing VCAM-1 expression in osteoarthritic synovial lining, an effect that was abolished in the presence of an α6β1 or αVβ5 integrin neutralizing antibody. CCN4 also increases activation of protein kinase C (PKCδ), JNK, AP-1 and Syk (Spleen tyrosine kinase) proteins, all of which are necessary for CCN4-mediated VCAM-1 upregulation [206]. Based on literature evidence, CCN4 elicits diverse and sometimes opposite functional effects depending on the cell-type and the spatial expression pattern within the musculoskeletal system. Another study conducted in 304 postmenopausal Japanese women indicates that genetic variations such as single nucleotide polymorphism in CCN4 gene locus has been linked with spinal osteoarthritis determined by radiographical observations such as disc space narrowing, endplate sclerosis and osteophyte formation, again highlighting its therapeutic utility as a novel diagnostic biomarker [207].

## Conclusion and future directions

**Fig. 3:**
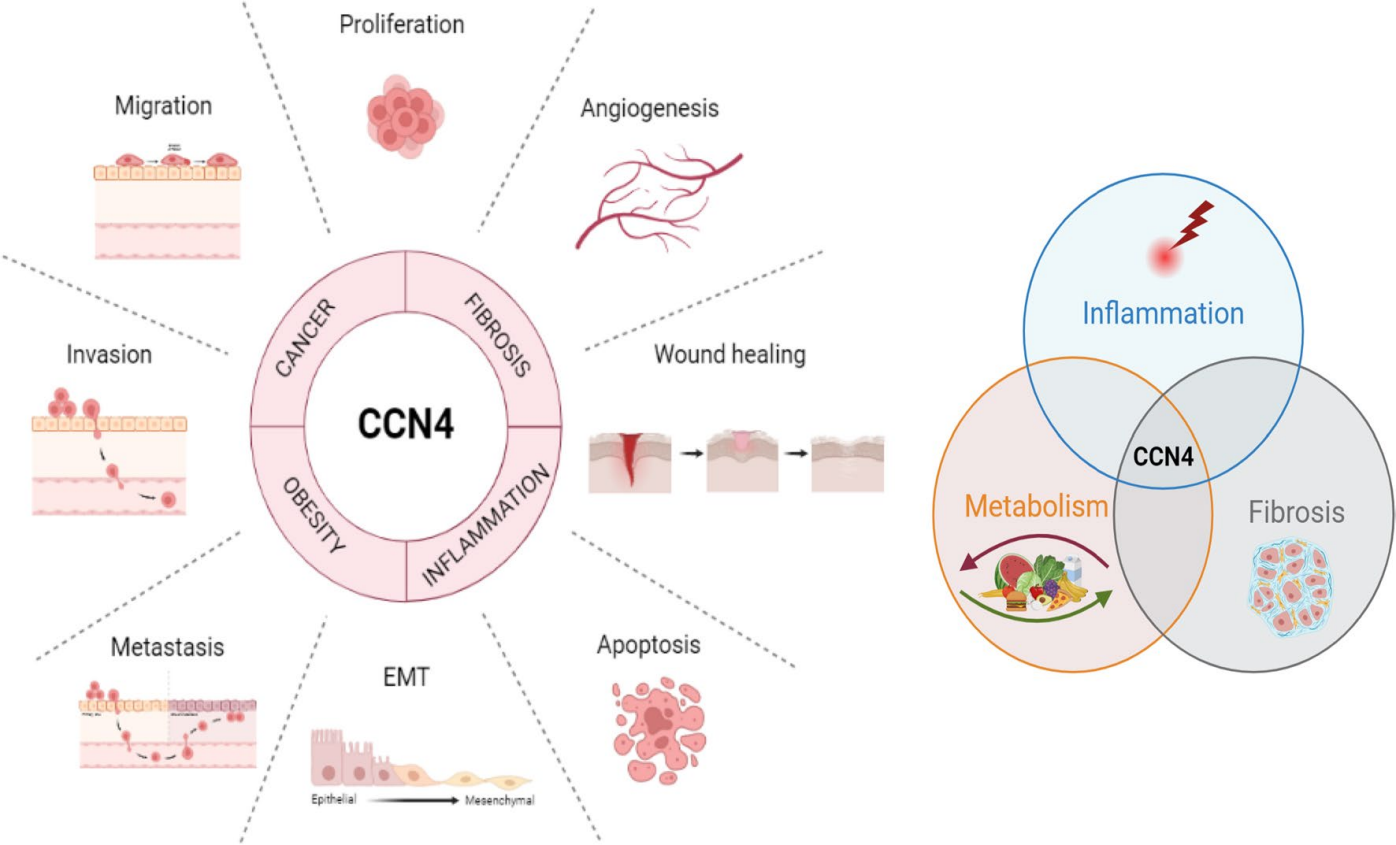
Functional effects of CCN4 in different diseases. CCN4 drives many cellular processes such as cell-proliferation, migration, invasion, epithelial to mesenchymal transition (EMT), apoptosis, wound healing, repair, and angiogenesis. Some of these functional effects are overlapping amongst a diverse array of pathological conditions like cancer, fibrosis, obesity, and inflammatory diseases. The image was created with BioRender.com

From the literature summarized herein, aberrant CCN4 expression is highly correlated with adverse clinical outcomes and demonstrates the significant role of CCN4 in diverse pathophysiological conditions, such as cancer, fibrosis, metabolic disorders, and arthritis. Altered CCN4 expression may not be the only factor controlling vast array of cellular functions, such as cell proliferation, migration, invasion, and wound healing, but it could be modulating and working in conjunction with other mitogenic signaling molecules, growth factors and inflammatory mediators in driving pathogenesis, as shown in Fig. 3.

As more translational studies unearth the CCN4-dependent molecular mechanisms, it is highly likely that CCN4 could be involved in the progression of many more undiscovered co-morbid pathologies. However, the slow growing research on CCN family is largely due to some major challenges in the field that remains to be addressed in future research. Besides the full-length CCN4 protein, identifying the domain specific function is crucial to understand the contribution of the variable linker in CCN biology. Development of domain specific detection tools for human and murine biological tissues or fluids is crucial to understand the tissue specific degradome/cleavage pattern of CCN4 in clinical models. In conjugation with protein–protein interaction studies, researchers could possibly identify functionally active domain(s) for monoclonal antibody mediated targeted therapy. Furthermore, an indirect approach could also be utilized by targeting the key proteolytic enzyme to prevent CCN4 cleavage and subsequently the formation of functionally active single or multi-domain structures. The proportion and expression kinetics of these multi-modular truncated CCN4 variants could reveal the functional redundancy and/or diverse effects in various diseases. Novel tools and reagents are also required to carefully assess the tightly regulated spaciotemporal expression and half-life of these individual or multi-domain structures which yet remains imperative to uncover its holistic biological significance. In addition, CCN4 signaling in diverse cell/tissue specific context still remains understudied. Multi-omics-based approaches at both transcript (RNA seq) and protein level (Proteomics) could be utilized to unravel other CCN4-dependent downstream targets and/or pathways. Also, immunoassays for multi-analyte profiling could further shed light on CCN4-dependend inflammatory secretome-signature. Furthermore, homo- or hetero-multimerization within CCN family members adds to the preexisting complexities. In addition, identifying the cognate receptor/s for CCN family members is important.

Another major challenge is to study crosstalk between CCN family of proteins. One of the major gaps in the current research is how other CCN members interact and modulate CCN4 in a synergistic or antagonistic manner in a pathological context. Some CCN family members have opposing effects with respect to CCN4. A bispecific targeting approach could also be utilized to target antithetical or synergistic CCN members to attain better therapeutic outcomes. However, given the high degree of structural and sequential homology, it is of paramount importance to first understand why some CCN members have opposite functional effects in a disease specific context. Minor changes in the amino acid sequence can bring about huge variations in the overall protein folding and 3-dimensional structure, affecting the surface charge and substrate binding potency, which could be utilized for targeted strategies. Given that CCN4 drives diverse cellular processes in a tissue-specific context, a comprehensive analysis encompassing the molecular network between all the CCN family members remains indispensable. Taken together, the results from the literature reviewed here suggest that CCN4 plays a critical role in the development and progression of diverse pathologies and is emerging as a promising candidate with therapeutic potential yet untapped.

## Data Availability

Not applicable.

## Abbreviations

ADAM28: Disintegrin and metalloproteinase domain-containing protein 28
Akt: Protein kinase B
AP-1: Activator protein-1
AT-II: Type II alveolar epithelial cells
BMI: Body mass index
BMP: Bone morphogenetic protein
CCL: Chemokine C–C motif ligand
CCN4: Cellular communication network factor 4
CD: Cluster of differentiation
CKD: Chronic kidney diseases
CT: C-terminal cysteine knot
CTGF: Connective tissue growth factor
CXCL: Chemokine C-X-C motif ligand
Cyr6: Cysteine-rich protein 61
DN: Diabetic nephropathy
ECM: Extracellular matrix
EGFR: Epidermal growth factor receptor
EMT: Epithelial to mesenchymal transition
ERK: Extracellular signal-regulated kinase
FAK: Focal adhesion kinase
FGF: Fibroblast growth factor
FN1: Fibronectin
GLUT1: Glucose transporter 1
HIF-1α: Hypoxia inducible factor 1 alpha
ICAM-1: Intracellular adhesion kinase 1
IFN-γ: Interferon gamma
IGFBP: Insulin-like growth factor binding protein like domain
IL-6: Interlukin-6
IPF: Idiopathic pulmonary fibrosis
IRS: Insulin receptor substrate 1
JNK: C-Jun N-terminal kinase
LRP: Low-density lipoprotein receptor-related proteins
MEK: Mitogen-activated protein kinase kinase
miRNA: MicroRNA
MMP: Matrix metalloproteases
MRTF: Myocardin-related transcription factor
mTOR: Mechanistic target of rapamycin
NF-κB: Nuclear factor kappa B
NOV: Nephroblastoma overexpressed
OA: Osteoarthritis
PI3K: Phosphoinositol 3-kinase
PKC: Protein kinase C
PPARγ: Peroxisome proliferator-activated receptor gamma
PTM: Post-translational modification
RA: Rheumatoid arthritis
TGF-β: Transforming growth factor: β
TLR: Toll-like receptor
TSP1: Thrombospondin-homology type 1 repeat
VCAM-1: Vascular cell adhesion protein 1
VEGF: Vascular endothelial growth factor
VWC: von Willebrand factor C repeat
WISP1: Wnt-1 induced secreted protein-1
αSMA: α-Smooth muscle actin
